# 

**Published:** 1890-02

**Authors:** 


### Society Meetings.

---

### THE MEDICAL SOCIETY OF THE STATE OF NEW YORK.

Provisional programme of papers for the eighty-fourth annual meeting in the City Hall, at Albany, on Tuesday, Wednesday and Thursday, February 4th, 5th, and 6th, 1890.

### OFFICERS OF THE SOCIETY.

*President*—Daniel Lewis, New York. *Vice-President*—Alfred Mercer, Syracuse. *Secretary*—F. C. Curtis, Albany. *Treasurer*—C. H. Porter, Albany.

*Committee on Business*—George Henry Fox, Henry Flood, Herman Bendell. *Committee of Arrangements*—Samuel B. Ward, Edward L. Partridge, C. S. Merrill *Committee on By-Laws*—F. A. Castle, A. R. Simmons, F. C. Curtis. *Committee on Hygiene*—E. V. Stoddard, A. N. Bell, W. B. Brown, J. P. Creveling, W. H. Bailey, W. C. Bailey, E. F. Brush. *Committee on Legislation*—D. B. St. John Roosa, B. Pillsbury, M. J. Lewi. *Committee on Ethics*—A. Jacobi, A. Matthewson, H. R. Hopkins. *Committee on Prize Essays*—G. F. Shrady, F. P. Foster, Eugene Beach. *Committee on Publication*—F. C. Curtis, J. O. Roe, O. B. Douglas, C. H. Porter.

### FIRST DAY—TUESDAY. MORNING SESSION—9.30.

President's inaugural address, appointment of committees, executive business.

Papers.—"The Use of Belladona in Spasmodic Contractions of the Neck during Labor," S. S. Cartwright, Roxbury. "The Time-Element in Saving the Perineum," Robert L. Dickinson, Brooklyn. "A Case of Extra-uterine Pregnancy," H. De V. Pratt, Jr., Elmira. "What are the Rational Limitations of Intra-uterine Therapeutics, Andrew F. Currier, New York. "Remarks on the Use of the Uterine Curette," Walter B. Chase, Brooklyn. "Cancer of the Body of the Uterus," H. C. Coe, New York; "The Indications for Operation in Disease of the Uterine Appendages," Egbert H. Grandin, New York. "Seven Consecutive, Successful Cases of Intubation of the Larynx for Diphtheritic Croup," Wm. Hailes, Jr., Albany.

### AFTERNOON SESSION—2.30.

"On the Treatment of Eczema in Elderly People," L. Duncan Bulkley, New York. "An Original, Simple, and Effective Method for Treatment of Scabies," Samuel Sherwell, Brooklyn. "On a Case of Leprosy Apparently Cured," George Henry Fox, New York. "Acute Dacryocystitis," David Webster, New York. "Spectra of Blood in Certain Eye Diseases," Lucien Howe, Buffalo. "Cataract," Francis Valk, New York. "A Case of Meningocele Resembling Degeneration of the Lachrymal Sac," W. F. Mittendorf, New York. "Purulent Ophthalmia," J. A. Andrews, New York.

EVENING SESSION—7.30.

"The Thomas Hip Spint," J. F. Ridlon, New York. "A Ready Method for Counter-traction at the Knee," Henry Ling Taylor, New York. "The Pendent Limb in the Treatment of Joint Diseases of the Lower Extremity," A. B. Judson, New York. "The Prevention of Tuberculous Bone Lesions," V. P. Gibney, New York. "The Question of What Produces and What Prevents Anchylosis of Joints," A. M. Phelps, New York. "A Statistical Paper on Club Foot," W. R. Townsend, New York.

SECOND DAY—WEDNESDAY. MORNING SESSION—9.30.

Executive Business.

"Fever and its Treatment," Alfred L. Loomis, New York. "A Case of Uremic Convulsions in New-born—Recovery," A. Hutchins, Brooklyn. "Functional Abuminuria, with Three Years' Observation of a Case," Eli H. Long, Buffalo. "The Importance of a State or National Registration of Births," Alex. Hadden, New York. "Isolation of Consumptives," P. H. Kretzschmar, Brooklyn. "The Production of Tubular Breathing in Solidification and Other Conditions of the Lungs," Charles Cary, Buffalo. "The Physiological, Pathological, and Psychological Bearings of Sex," Sam'l S. Wallian, New York. "A Study of Alcoholism in Bellevue Hospital," C. L. Dana, New York. "Early Diagnosis of Tubercular Meningitis," W. P. Northrup, New York. "Recent Lunacy Legislation Affecting the General Practitioner," E. H. Howard, Rochester. "The Prompt Recognition and Treatment of Syphilis of the Brain," J. Leonard Corning, New York.————, E. F. Brush, Mount Vernon.

AFTERNOON SESSION—2.30.

Address on "Surgery," Alfred Mercer, Syracuse. "The Diagnosis and Treatment of Fractures Through the Anatomical Neck of the Humerus," C. A Powers, New York. "Report of Five Cases of Supra-pubic Cystotomy for Tumors of the Bladder and Vesical Calculi," A. Vander Veer, Albany. "Reflections upon Some Cases of Nephro-lithotomy," E. L. Keyes, New York. "A Rarely Described but Common Form of Cystitis in Women," Robert T. Morris, New York. "An Extension of Splint for the Treatment of Fractures of the Leg, the Arm, and Fore-Arm," P. R. Furbeck, Gloversville. "A History of 250 Cases of Excision of Superior Maxilla," J. D. Bryant, New York. "Treatment of the Diseases of the Rectum based upon Anatomical Study, Seneca D. Powell, New York.

EVENING SESSION.

Anniversary Address by the President, eight o'clock. Annual dinner of the Society, nine o'clock.

THIRD DAY—THURSDAY. MORNING SESSION—9.30.

"Methods of Examining the Post-Nasal Pharynx and Surgical Treatment of Diseases of that Cavity," C. C. Rice, New York. "A Case of Papilloma of the Vocal Chords," Charles N. Cox, Brooklyn. "The Prevention of Tubercular Laryngitis," B. F. Westbrook, Brooklyn. "Nasal Intubation," D. H. Goodwillie, New York. "Nasal Epilepsy," John O. Roe, Rochester. "Some Practical Suggestions Concerning Antrum Disease," F. H. Bosworth, New York. "Chronic Nasal Catarrh; Its Causes, Course, and Cure," O. B. Douglas, New York. "Naso-Pharyngeal Epithelioma," Sidney Allan Fox, Brooklyn. "The Etiology and Radical Treatment of Nasal Myxomata," W. C. Jarvis, New York.

West 23d street, New York. This hand-book combines the conveniences of a diary with those of a manual, and is of great benefit to the practising physician. This edition has been thoroughly revised—containing all the recent discoveries in materia medica and therapeutics, together with the new remedies which of late have come in vogue.

It is bound in English morocco, pocket-book form, and is retailed at $1.50.

---

### BOOKS AND PAMPHLETS RECEIVED.

The National Medical Dictionary, including English, French, Italian and Latin technical terms used in Medicine and the Collateral Sciences, and a Series of Tables of useful data. By John S. Billings, A. M., M. D., LL. D., Edin. and Harv., D. C. L. Oxon. Vol. I., A to J. Vol. II., K to Z. Philadelphia: Lea Brothers & Co. 1890.

A Hand-Book of Diseases of Women; Including Diseases of the Bladder and Urethra. By Dr. F. Winckel. Authorized Translation. Edited by Theophilus Parvin, M. D. Second edition. Revised and enlarged with 150 illustrations. Philadelphia: P. Blakiston, Son & Co., 1012 Walnut street. 1889.

A Guide to the Diseases of Children. By James Frederic Goodhart, M. D., F. R. C. P. Re-arranged, revised, and edited by Louis Starr, M. D. Second American, from the third English edition, with numerous Formula and Illustrations. Philade'phia: P. Blakiston, Son & Co. 1889.

Quiz-Compends. No. 4. A Compend of Human Physiology. Especially adapted for the use of Medical Students. By Albert P. Brubaker, A. M., M. D. Fifth edition. Revised and enlarged. With new illustrations. Philadelphia: P. Blakiston, Son & Co. 1889.

Through the Ivory Gate. Studies in Psychology and History. By William W. Ireland, M. D., Edin. New York: G. P. Putnam's Sons. Buffalo: Peter Paul & Bro.

Ennucleation of Tuberculous Glands. By Thos. W. Kay, M. D., Scranton, Pa. Reprint from the *Medical Register*, February 9, 1889.

Further Observations on Malarial Keratitis. By Charles J. Kipp, M. D., Newark, N. J. Reprint from the Transactions American Ophthalmological Society. 1889.

Persistent Headaches, and How to Cure Them. By Julian J. Chisholm, M. D., Baltimore, Md. Reprint. Baltimore: Guggenheimer, Weil & Co., printers.

Report of the Commissioner of Education for the Years 1887-88. Washington: Government Printing Office. 1889.

A Malignant Tumor in an Umbilical Hernial Sac, with Remarks on the Etiology of Cancer. By Daniel Lewis, M. D. Reprint from the *Medical Record*, Oct. 12, 1889.

Erythroxylon Coca; Its value as a Medicament. By Marc Laffont, M. D., Paris. Reprint from the New York *Medical Journal*.

Misplacements of the Uterus. By Mary A. Dixon Jones, M. D., Brooklyn, N. Y. Reprint from the Pittsburgh *Medical Review.*

Some Physiological Facts bearing on the Production of the Nasal Vowels. By B. Loewenburg, M. D., Paris and Berlin. Reprint from the *Medical Press and Circular*, 1889. London: Bailliere, Tindall & Cox, publishers.

The Surgical Treatment of Volvulus. By N. Senn, M. D., Ph. D. Reprint from the *Medical News.*

A Digest of Twenty Years' Experienee in the Treatment of Uterine Cancer, including 367 Operations by Galvano-Cautery. By John Byrne, M. D., M. R. C. S. E. Reprint from the American Gynecological Society's Transactions, Vol. XIV., 1889.

Homeosis. By B. Fincke, M. D., Brooklyn, N. Y. Reprint from the *Journal of Homeopathics.*

Sanitary Entombment; the Ideal Disposition of the Dead. By the Rev. Charles R. Treat. Reprint from *The Sanitarian*, December, 1889.

Dr. Kállay's Artzlicher Almanach for 1890. Eleventh year. Vienna: Wilhelm Braumuller & Son. 1890.

Higher Medical Education and How to Secure It. The Annual Address before the Alumni Association of the University of Maryland. By Richard H. Lewis, M. D., of Raleigh, N. C.

The American Academy of Medicine; its Objects; its Signs of Promise and its Obstacles; its Field of Work; and Some Suggestions Looking to an Increase in its Efficiency. By Leartus Connor, M. D., A. B., Detrioit, Mich.

The Preferable Climate for Consumption. By Charles Denison, A. M., M. D. Reprint from the Transactions of the Ninth International Medical Congress. Vol. V. 1887. By order of the Legislature of Colorado.

Cornell University, College of Agriculture. Bulletin of the Agricultural Experiment Station. Agricultural Department. XIII. I. On the Deterioration of Farm Yard Manure by Leaching and Fermentation. II. On the Effect of a Grain Ration for Cows at Pasture. XIV. I. On the Strawberry Leaf Blight. II. On Another Disease of the Strawberry. December, 1889. Published by the University, Ithaca, N. Y.

Twenty-first Annual Report of the Trustees of the Willard Asylum for the Insane, for the year 1889. Albany: James B. Lyon, State Printer.

Third to the Sixth Annual Reports of the Buffalo Hospital of the Sisters of Charity, including the Emergency Hospital. November, 1885, to November, 1889. Buffalo: Baker, Jones & Co. 1889

Annual Report of the Rhode Island Hospital. 1889.

Lawrenceville School. John C. Green, Foundation. 1889-1890. The Princeton Press.

Thirteenth Annual Report of the Buffalo Eye and Ear Infirmary. Published by order of the Trustees. 1889.

State Board of Health Bulletin. Nashville, Tenn. December 20, 1889.

Monthly Bulletin of the New York State Board of Health. November, 1889.

Health in Michigan. December, 1889.

Weekly Abstract of Sanitary Reports. U. S. Marine Hospital Service. Vol. IV., Nos. 51 and 52. Vol. V., Nos. 1, 2, 3, and 4.

### Literary and Other Notes.

OUR acknowledgments are due to the following named houses for handsome and useful calendars for 1890: Parke, Davis & Co., Detroit; E. B. Treat, New York (The Don't Forget It Calendar, price 20 cents); F. D. Van Horen, New York; and the Davidson Rubber Company, Boston.

---

THE holiday number of the *Trained Nurse* (Lakeside Publishing Company, Buffalo, N. Y.) contains much of interest, and is handsomely printed. The scheme of a proposed organization, to be known as the New York State Trained Nurses' Association, is published in this number, which will prove of importance to all concerned. We see no good reason why the nurses should not form themselves into organizations for mutual improvement, and heartily wish them success in everything that may contribute to that end.

---

THE NORTH CAROLINA MEDICAL JOURNAL, one of our valued exchanges, appears in an improved form for 1890. This journal is making a strong claim for extended favor from the profession, not merely in North Carolina, but beyond the limits of that sturdy commonwealth. We wish it the full measure of success that it so well merits.

---

BELFORD'S MAGAZINE for January, 1890, is an attractive number, containing, among other articles of interest, two from the pen of the late Jefferson Davis: one on Andersonville and Other War Prisons, part i., and the other An Autobiography. The other papers, besides the stories, discuss questions of politics, economy, and social problems. The price of this magazine is so reasonable—$2.50 a year, single numbers 25 cents—and its appearance so attractive, that it cannot fail to receive an ample and deserving support.

---

THE DETROIT FREE PRESS SOUVENIR for 1890 is a handsome specimen of the printer's and engraver's arts. It is sent free to all who forward a year's subscription to the weekly edition at any time within twelve months from November 1, 1889. We will furnish the *Weekly Free Press*, including the souvenir, and the BUFFALO MEDICAL AND SURGICAL JOURNAL for 1890 for $2.50. The regular price of the two is $3.00.

